# Biotechnology Applications of Tethered Lipid Bilayer Membranes

**DOI:** 10.3390/ma5122637

**Published:** 2012-12-07

**Authors:** Joshua A. Jackman, Wolfgang Knoll, Nam-Joon Cho

**Affiliations:** 1 School of Materials Science and Engineering, Nanyang Technological University, 50 Nanyang Avenue, Singapore 639798, Singapore; E-Mail: josh0018@e.ntu.edu.sg; 2 Centre for Biomimetic Sensor Science, 50 Nanyang Drive, Singapore 637553, Singapore; E-Mail: wolfgang.knoll@ait.ac.at; 3 Austrian Institute of Technology (AIT) GmbH, Donau-City Str.1, Vienna 1220, Austria

**Keywords:** tethered lipid bilayer, supported lipid membranes, ion channel, stochastic sensing, cytochrome c, electrochemical impedance spectroscopy, fluorescence recovery after photobleaching, surface plasmon resonance, quartz crystal microbalance, two-dimensional surface-enhanced IR absorption spectroscopy

## Abstract

The importance of cell membranes in biological systems has prompted the development of model membrane platforms that recapitulate fundamental aspects of membrane biology, especially the lipid bilayer environment. Tethered lipid bilayers represent one of the most promising classes of model membranes and are based on the immobilization of a planar lipid bilayer on a solid support that enables characterization by a wide range of surface-sensitive analytical techniques. Moreover, as the result of molecular engineering inspired by biology, tethered bilayers are increasingly able to mimic fundamental properties of natural cell membranes, including fluidity, electrical sealing and hosting transmembrane proteins. At the same time, new methods have been employed to improve the durability of tethered bilayers, with shelf-lives now reaching the order of weeks and months. Taken together, the capabilities of tethered lipid bilayers have opened the door to biotechnology applications in healthcare, environmental monitoring and energy storage. In this review, several examples of such applications are presented. Beyond the particulars of each example, the focus of this review is on the emerging design and characterization strategies that made these applications possible. By drawing connections between these strategies and promising research results, future opportunities for tethered lipid bilayers within the biotechnology field are discussed.

## 1. Introduction

From hosting transmembrane proteins involved in signal transduction pathways to regulating what enters and exits a cell, phospholipid membranes represent a critically important interface within biological systems [1,2,3,4]. Since natural cell membranes are complex and possess many different components [5], a range of simplified model systems, such as lipid vesicles in solution and black lipid membranes, have been established to mimic the fundamental architectural element of the membrane: the lipid bilayer. More recently, there has been interest in developing model membranes on solid supports [5,6,7]. Compared to other model systems, supported membranes represent a particularly advantageous platform, because they are physically stabilized and amenable to characterization by a wide range of surface-sensitive techniques [8]. In principle, supported membranes are based on either a planar lipid bilayer or a layer of intact vesicles. Several excellent references are available that provide a comprehensive overview of solid-supported model membrane systems, including their design and characterization [9]. Important design criteria include high electrical sealing and the ability to host transmembrane proteins. With biotechnology applications now emerging, another increasingly important requirement is air stability. An overview of different types of model membrane systems is presented in Table 1.

**Table 1:** Overview of different model membrane systems.

Model System	Description	Surface-Sensitive Characterization	Electrical Sealing	Membrane Proteins	Air Stability
Lipid vesicles	Freely diffusing vesicles suspended in aqueous solution	No	Yes	Yes	No
Black lipid membrane	Lipid bilayer spans an open aperture	No	Yes	Yes	No
Supported lipid bilayer	Two-dimensional lipid bilayer physisorbed on solid support	Yes	Yes	No	Yes
Tethered lipid bilayer	Planar bilayer immobilized on solid support or soft cushion	Yes	Yes	Yes	Yes
Intact vesicle layer	Layer of intact vesicle physisorbed on solid support	Yes	No	Yes	No
Tethered vesicles	Intact vesicles immobilized to solid support or planar bilayer	Yes	No	Yes	No

In this review, the focus is on a particular class of model membranes called tethered lipid bilayers. As shown in Table 1, the tethered bilayer platform is uniquely capable among the different types of model membranes to meet the requirements for fundamental and applied science. Inspired by planar lipid bilayers on solid supports, tethered bilayers are rugged derivatives that are immobilized to the support and are, thus, popular sensing platforms for biotechnology applications. A principle advantage of tethered lipid bilayers is their long-term stability on the order of days, weeks or even months. At the same time, tethered bilayers can also recapitulate basic properties of biological membranes, including two-dimensional fluidity in the liquid crystalline phase and exposure to aqueous reservoirs on each side of the bilayer. While early fabrication of these platforms used the Langmuir-Blodgett deposition technique for monolayer transfer [10], robust self-assembly methods are commonly employed today for technical simplicity and high reproducibility. 

**Figure 1:**
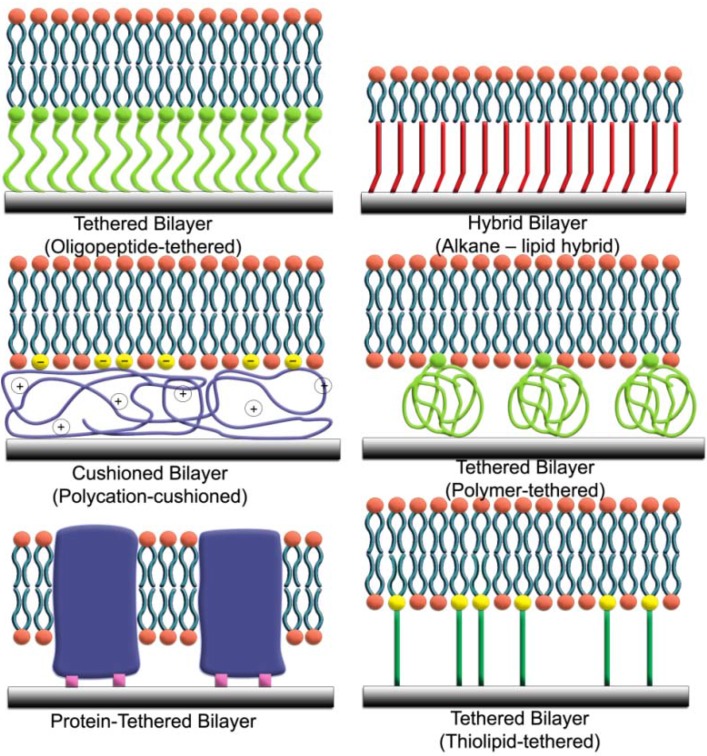
Design strategies for tethered lipid bilayers on solid supports. A wide range of tethering units can support the assembly of tethered lipid bilayers on solid supports.

An important design criterion for tethered lipid bilayers is the tethering unit, which connects the membrane to the underlying solid support. Examples of commonly used tethers for supporting planar lipid bilayers are presented in Figure 1 below. Depending on the application, there are advantages and disadvantages to each type of tether. First-generation versions employ an alkanethiol tether that is covalently bonded to thin films of metals, such as gold [11] and mercury [12]. Alkanethiol monolayers readily form on these metals from thiols dispersed in ethanol because of the strong affinity of metals for sulfur atoms [13,14]. The resulting self-assembled monolayer (SAM) has hydrophobic surface properties arising from the alkane chains, which mimic lipid chains. The adsorption of lipid vesicles onto the SAM promotes the formation of a hybrid bilayer, which consists of a lower leaflet of alkanethiols and an upper leaflet of phospholipids [11,15]. Although the hybrid bilayer has many useful properties and provides the general framework for the tethered bilayer field, there is no aqueous layer between the lower leaflet of the bilayer and the metal surface. This technical limitation has prompted the development of tethered bilayers with spacer regions between the lower leaflet of the bilayer and the solid support.

An alternative approach focuses on the tethering of macromolecular spacers, such as polymer chains [6,16], polyelectrolyte layers [17], carbohydrates or peptides [18,19,20], to the solid support in order to provide a soft cushion for the bilayer. Lipid vesicles can then fuse upon the layer of tethered macromolecules to form a planar lipid bilayer that is separated from the solid support by at least several nanometers. This separation distance is sufficient to host transmembrane proteins and to support ion transport across the membrane. However, the lipid bilayer is not itself tethered, but rather supported upon the tethered layer, and this design limits the ruggedness of the model membrane. In addition, such bilayers do not have sufficient electrical sealing properties for quantitative characterization of ion channel activities. To address these issues, new tethering elements have emerged that combine the utility of thiol groups, macromolecular spacers and phospholipids. 

Termed thiolipids, the central feature of these tethers is a hydrophilic spacer that is capped with a functional group for covalent immobilization [21,22,23,24,25]. The other end of the spacer is linked to the hydrophilic head of a phospholipid. Like with SAMs of alkanethiols, monolayers or sub-monolayers of thiolipids can self-assemble on noble metals in organic solvents, and the kinetics of this process depends on several parameters, including incubation time, thiolipid type and concentration, the presence of short dilution thiols and substrate properties. Again, the functionalized substrate presents a hydrophobic surface that promotes the fusion of lipid vesicles to form a tethered lipid bilayer. However, in this case, the spacer function of the thiolipid separates the lower leaflet of the bilayer from the solid support to enable functional reconstitution of membrane-associated proteins. Taken together with excellent electrical sealing properties and long-term stability, the structure of thiolipid-based tethered bilayers makes them excellent platforms for studying ion channels, pore-forming toxins and transmembrane proteins. While metal thin films have advantageous properties for electrochemical and plasmonic optical applications, there is also interest in developing thiolipid-based tethered bilayers on silicon oxide for microelectronics, and a similar approach using silane chemistry has been developed for this case [26]. Likewise, phosphonic acid esters have been incorporated into tethers for immobilization on aluminum oxide [27].

In a broader context, as the molecular engineering of tethered lipid bilayers continues to advance with the integration of synthetic strategies and lessons from nature, there are increasing opportunities for translation of basic science into biotechnology applications. The rest of this review introduces several examples of emerging capabilities within the tethered lipid bilayer field. With each example highlighting a particular component of tethered lipid bilayers, such as characterization methods or ion channel engineering, the collective picture generated from these examples provides a framework to understand how tethered bilayers are moving closer to becoming widespread in various technologies. 

## 2. Characterization Methods

Historically, freestanding lipid membranes were monitored by light microscopy, with membrane thinning inferred as a decrease in reflectance [28]. Hence, these early model systems were referred to as black lipid membranes. By contrast, a wealth of surface-sensitive analytical techniques is now available for quantitative characterization of solid-supported model membranes. Sensor technologies based on optical, acoustic, electrochemical or fluorescent measurement principles provide complementary methods for determining the structural and functional properties of tethered lipid bilayers. One of the most important surface-sensitive techniques for tethered bilayers has been surface plasmon resonance (SPR) imaging, which can measure the optical thickness of organic thin films [29]. By monitoring the change in film thickness as a function of time, SPR imaging and more recently applied tools, such as the quartz crystal microbalance (QCM), can follow the self-assembly of tethered lipid bilayers in a label-free format [30]. After self-assembly of the tethered bilayer, direct measurement of membrane thickness using methods developed for atomic force microscopy (AFM) can confirm morphology [31].

In addition, surface-sensitive characterization methods can measure the functional properties of tethered lipid bilayers. Fluorescence microscopy is useful for determining the homogeneity of fluorescence-doped planar bilayer systems, and fluorescence recovery after photobleaching (FRAP) can be applied to measure the lateral diffusion of phospholipids within such membranes. As sensing platforms, tethered lipid bilayers must also have sufficient electrical sealing properties. Impedance spectroscopy is an excellent tool to characterize the electrical properties of the membrane, including resistance and capacitance. More information about the full breadth of surface-sensitive characterization tools for model membranes is comprehensively detailed in several excellent reviews [32]. With all of these tools widely utilized for studying model membranes, their capabilities are continually being used in novel ways to understand how the structure of a tethered bilayer is linked to its function.

### 2.1. Functional Analysis of Membrane-Associated Peptides

The general design principles of thiolipid-based tethered bilayers are particularly well-suited for electrochemical applications. When tethered bilayers are constructed on conductive metal surfaces, such as gold or mercury, the solid support can serve as the active electrode and an ionic reservoir interfaces the electrode with the membrane transducer that converts biological signals into electrical signals. As such, the structural properties of the hydrophilic spacers that tether the membrane to the support influence their sensing capabilities. Particularly promising work on this subject has been reported for oligoethyleneoxy spacers [21,22,23,24,25,33,34,35,36]. Compared to alkanethiols, the spacer region of these molecules consists of bulky oligoethyleneoxy groups, which limit SAM packing density and, in turn, increase the size of the ionic reservoir. Among the different thiolipids within this class are 2,3-di-O-phytanyl-sn-glycerol-1-tetraethylene glycol-D,L-α-lipoic acid ester (DPTL) and 2,3-di-O-phytanyl-sn-glycerol-1-hexaethylene glycol-3’-mercaptopropyl ether (DPHT). DPTL has a tetraethyleneoxy spacer and anchors to solid supports by two metal-sulfur bonds, whereas DPHT has a longer hexaethyleneoxy spacer and uses only one metal-sulfur bond for immobilization.

**Figure 2:**
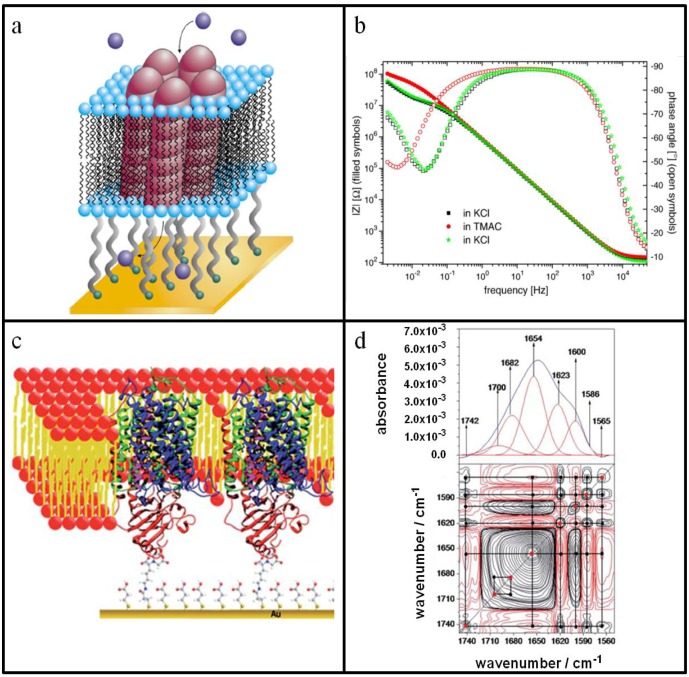
Evaluation of membrane-active proteins using surface-sensitive techniques. (**a**) Pictorial representation of a functional ion channel comprised of five M2 peptides that self-assembles within a tethered lipid bilayer on a gold surface. In this particular case, the lower leaflet of the bilayer exclusively consists of tethered anchor lipids. (**b**) Electrochemical impedance data measures the blocking effect of TMA on functional properties of tethered DPTL lipid bilayer with embedded M2 pentameric ion channels at 0 V potential (*vs*. AgCl reference). Closed and open symbols depict the impedance and phase shift, respectively. (**c**) Pictorial representation of the protein-tethered lipid bilayer based on the covalent tethering of cytochrome c oxidase from *R. sphaeroides*. (**d**) 2D correlation maps of activated cytochrome c oxidase for synchronous signal in the amide I region from SEIRA difference spectra reduced—fully oxidized for CcO immobilized via His-tag on subunit II. **Top panel:** 1D-SEIRA difference spectra (taken at −700 mV) as an example for the deconvolution. **Bottom Panel:** Correlation maps of a series of spectra as a function of potential. The potential applied was 900 mV *vs.* SHE for the fully oxidized state and varied from 500 mV to −700 mV in 100 mV steps for reduced states. Parts A and B are adapted with permission from [37]. Copyright 2007 Elsevier. Parts C and D are reproduced by permission of The Royal Society of Chemistry from [38]. Copyright 2011 The Royal Society of Chemistry.

Based on these differences, Vockenroth *et al.* sought to understand how spacer architecture in tethered bilayers influences functional incorporation of M2 peptides that are derived from the membrane-spanning domain of the acetylcholine receptor [37]. Membrane-associated M2 peptides self-assemble to form pentameric oligomers that selectively transport small monovalent cations, such as Na^+^ and K^+^, across the bilayer (Figure 2a). By using electrical impedance spectroscopy (EIS), the effects of membrane-peptide interactions can be detected by changes in the electrical properties of the tethered bilayer system. EIS spectroscopic data is typically presented in Bode plots that express impedance and phase angle as functions of the applied frequency. Model fitting can then yield membrane resistance and the capacitance of the ionic reservoir between the solid support and the bilayer.

Figure 2b presents a Bode plot for a DPTL-tethered bilayer containing M2 peptides under different buffer conditions. In the presence of KCl buffer solution, the bilayer had an initial resistance of 3 MΩ·cm^2^. When the solution was exchanged to a buffer containing larger monovalent cations (tetramethylammonium, TMA^+^) that cannot pass through the pentameric pores, the resistance increased to 15 MΩ·cm^2^. This five-fold change in membrane resistance demonstrates that the functionalized tethered lipid bilayer maintains excellent sealing properties for a high signal-to-noise ratio. Likewise, the interfacial capacitance decreased by ~27%, because TMA^+^ ions cannot penetrate the bilayer and the spacer region, therefore, has fewer ions per electrode surface area. When the solution was re-exchanged to KCl buffer solution, the membrane resistance and interfacial resistance returned to the original values, confirming signal reversibility.

Similar trends in electrical properties of the system were observed for DPHT-tethered bilayers under the same set of buffer conditions. However, the EIS spectroscopic measurements also revealed differences in absolute values of membrane resistance and interfacial capacitance that correlated with the structural properties of the tethers. In the presence of KCl solution, the interfacial capacitance of the DPHT-tethered bilayer was ~32% greater than that of the DPTL-tethered bilayer. Since DPHT has a longer spacer than DPTL, the larger capacitance is attributed to greater ion flux. Interestingly, membrane resistance was identical for both types of tethers under conducting conditions (~3 MΩ·cm^2^ in the presence of KCl solution) but not under non-conducting conditions (~15 MΩ·cm^2^ for DPTL and ~35 MΩ·cm^2^ for DPHT in presence of TMACl solution). The authors suggested that this difference may be caused by different packing densities of DPTL *vs.* DPHT tethers, because they anchor to the gold substrate by different mechanisms. 

Although further studies are needed to explain this observation, such differences provide insight into how the model membrane platform can be optimized for biosensor applications. The DPHT-tethered bilayer has a greater signal-to-noise ratio than that of the DPTL-tethered bilayer, and this discrimination could be useful for developing electrical readouts with high sensitivity. On a broader scope, the study also underscores the importance of designing tethered bilayers that are suitable for the desired application. While thiolipid-based systems have proven capable to study small peptides and ion channels, such as valinomycin and gramicidin, they have proven less adept for characterizing the function of more complex proteins, especially those with large extramembranous domains or those which require conformational changes for activation [38]. As a result, new design strategies have been employed to host larger proteins in tethered lipid bilayers. 

### 2.2. Functional Analysis of Membrane-Associated Proteins

One of the most successful strategies for embedding large proteins within tethered bilayers focuses on the protein as the fundamental building block of the platform rather than the lipid bilayer. In the original method reported by Giess *et al.* [39], a solid support is functionalized with N-hydroxy succinimide (NHS) ester groups before coupling ion chelating nitroloacetic acid groups to the surface. Upon addition of a divalent cation, such as Cu^2+^ or Ni^2+^, His-tagged transmembrane proteins can be reversibly immobilized to the solid support based on a traditional method of reconstitution. The detergent-solubilized form of protein is first immobilized by chelation, and then phospholipid molecules replace the detergent molecules through *in situ* dialysis (Figure 2c). Two important benefits arise from this strategy. By varying the surface concentration of the chelating agent, the packing density of the tethered protein can be controlled, and there can be an optimal density for protein activity. Further, the tethered protein can be preferentially oriented towards the substrate depending on the location of the His-tag at the N- or C-terminus of the protein. As seen with the following example, the ability to control protein orientation offers the tethered bilayer platform a unique capability to study the electrochemistry of membrane-associated biological processes.

Cytochrome c oxidase (CcO) is a complex, multi-subunit protein that is involved in the respiratory electron transport chain, where it receives four electrons from reduced cytochrome c molecules, and transfers these electrons along a series of electron-accepting redox centers in order to convert one oxygen molecule into two molecules of water. When embedded in the tethered bilayer, EIS and cyclic voltammetry (CV) measurements demonstrate that CcO can transport electrons during the catalytic redox cycle. Depending on how the protein is oriented with respect to the surface electrode, direct electron transfer can occur under anaerobic conditions, and this capability enables quantitative kinetic analysis of the enzyme mechanism. Through early studies of direct electron transfer, it was suggested that CcO transitions from a non-activated to an activated conformational state after the enzyme passes through a number of redox cycles under aerobic conditions.

In order to gain insight into the structural changes that occur during the activation of CcO enzyme, Nowak *et al.* performed surface-enhanced infrared absorption (SEIRA) spectroscopy experiments across a range of potentials corresponding to reducing and oxidizing environments [40]. While one-dimensional SEIRA spectroscopy can be used to assign bands to different redox centers, such as Cu and heme, within the enzyme, there was no marked difference between the spectra collected for CcO in the non-activated *vs.* activated states (See Figure 2d, Top Panel for representative spectrum). By contrast, two-dimensional infrared spectroscopy (2D-IR) can determine the sequential order of conformational transitions as a function of perturbation, which in this case is the varying applied potential, using correlation maps. Diagonal peaks in the maps represent peaks that are identified in both perpendicular and parallel polarization 2D-IR spectra. These peaks provide information about backbone structure, whereas cross peaks off the diagonal line indicate inter- and intra-molecular interactions among functional groups. (See Figure 2d, Bottom Panel for a representative correlation map). 

Taken together, the molecular discrimination afforded by 2D-IR spectroscopy was able to identify strongly correlated conformational transitions at individual amino acid positions, which supports that the activated state of CcO undergoes a more global conformational change upon activation. In the activated state, CcO was found to assume a more condensed structure, presumably to facilitate efficient transfer of protons and electrons across the redox centers. Several key features from 2D-SEIRA spectroscopy support this conclusion, including a marked increase in α-helices *vs.* β-sheets in the enzyme. A higher degree of cooperativity among single transitions was also found in the activated state as the result of hydrogen bonding, hydrophobic interactions and dipole interactions. In addition, the number of amino acids identified through 2D-IR spectroscopy was greater in the activated *vs.* non-activated state, suggesting that the activated state assumes a more rigid form that is consistent with optimal activity. 

Interestingly, these findings not only confirm earlier X-ray crystallographic studies that proposed a significant conformational change of CcO upon reduction, but also link this conformational change to a specific mechanism of electron transfer. As such, the membrane-mimicking nature of tethered lipid bilayers provides a unique approach to understand the mechanism of enzymatic activities, particularly those which involve electrochemical processes. With the demonstrated capability to couple sensor electronics to biological transducers so that bioelectrical signals can be transmitted—in this case, electron transfers that occur as part of CcO enzyme activity—tethered lipid bilayers offer the potential to engineer artificial systems from biological components in order to create new classes of biosensors. A particularly compelling example of such technology is found in the class of ion channel biosensors.

## 3. Biotechnology Applications

### 3.1. Ion Channel Biosensors

#### 3.1.1. Influenza Virus Detection

**Figure 3:**
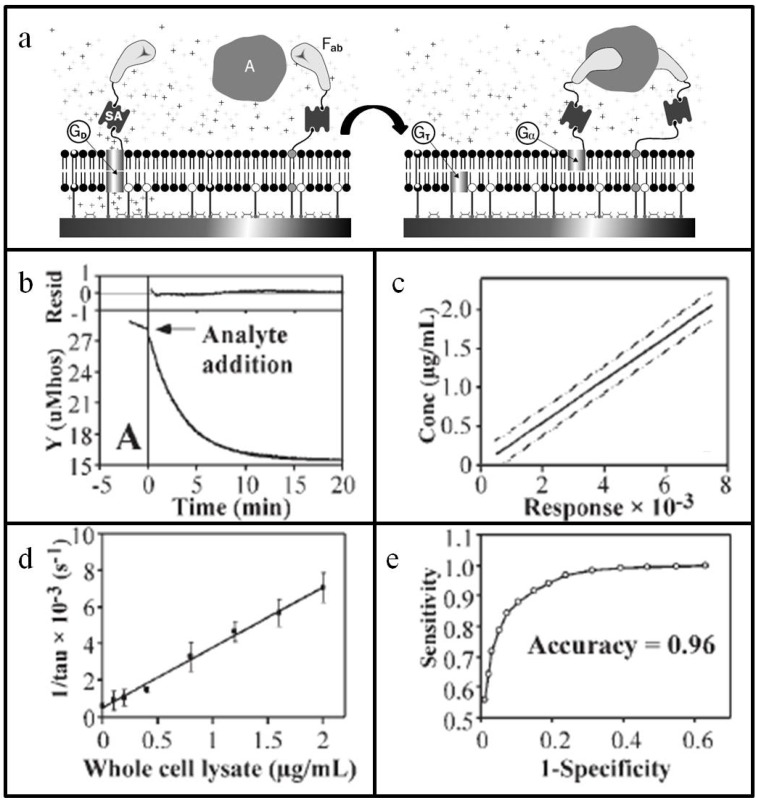
Tethered lipid bilayer for influenza virus detection. (**a**) Schematic of ion-channel switch (ICS) biosensor. Antibody fragments on lipid and gramicidin components of the biosensor cross-link the target analyte. This binding event abrogates transient formation of conducting gramicidin dimers and is recorded as a decrease in admittance; (**b**) Typical change in admittance as a function of time after addition of analyte; (**c**) Based on molecular engineering of the tethered bilayer, the response curve exhibits strong linearity across a wide concentration range; (**d**) The assay shows a low detection limit of 0.4 μg·mL^−1^ of total protein that is comparable to existing biological assays, albeit without sample processing; (**e**) Clinical simulations based on Receiver Operating Characteristic show that the assay readout yields 96% accuracy. Adapted with permission from [42]. Copyright 2005 Gen Publishing Inc.

The first example of a single-molecule experiment on an identified functional biomolecule was conducted over 40 years ago: the observation of current flow through a single ion-conducting channel formed by the peptide antibiotic gramicidin in a planar lipid bilayer [41]. Inspired by these early studies, Cornell *et al.* established a new class of biosensor that uses gramicidin ion channels embedded in the tethered bilayer as electrical switches [34]. Their approach—known as the ion channel switch (ICS) biosensor—measures the change in ion conductance through a population of transient gramicidin dimers within a tethered lipid bilayer upon binding of the target analyte (Figure 3a). Although analyte detection by the ICS biosensor is fundamentally simple, the particular approach is noteworthy, because it relies on molecular recognition elements for both high specificity and signal amplification. The lower leaflet of the bilayer consists of a combination of mobile and immobile (*i.e.*, covalently tethered) lipids along with tethered gramicidin monomers and a small fraction of functionalized bilayer-spanning tethered lipids. The headgroup of the functionalized lipid is conjugated by biotin-steptavidin linkage to an antibody fragment that recognizes the target analyte. In the upper leaflet of the bilayer, there are mobile lipids, as well as biotinylated, yet still mobile, gramicidin monomers. Via streptavidin coupling, the gramicidin monomers of the upper leaflet are also each affixed to another antibody fragment that recognizes the target analyte and is complementary to the antibody fragment attached to the bilayer-spanning tethered lipids. 

In the absence of analyte, the gramicidin monomers in the upper and lower leaflets transiently assemble to form dimers. Under an applied potential, this arrangement produces a steady population of conductive pores that allow ions to pass through the bilayer at a rate of up to a million ions per second, thereby generating a characteristic admittance signature. When the target analyte is introduced into the system, the antibody fragments on the bilayer-spanning, immobile lipids and on the biotinylated, laterally-diffusing gramicidin monomers eventually cross-link the analyte. The once-mobile gramicidin monomers of the upper leaflet become immobile and cannot assemble together with the immobile gramicidin monomers of the lower leaflet. The result is a drop in the admittance signal arising from the decrease in the population of conducting pores. 

Building on this framework, Lee *et al.* developed an ICS biosensor that can detect influenza virus, a widely circulating infectious disease that killed millions of people throughout the 20th century [42]. In order to create a specific assay, the antibody fragments embedded in the tethered lipid bilayer were derived from monoclonal antibodies that recognize the influenza nucleocapsid protein. For proof-of-concept studies, chicken cells were infected with influenza virus before the cells were lysed and the protein contents were extracted. The lysate was used as a target analyte in the assay and contained chicken egg protein and viral proteins, including the nucleocapsid protein. Importantly, not only was a drop in admittance from the gramicidin ion channels observed when the lysates were characterized with the tethered lipid bilayer, but the signal response occurred quickly on the order of minutes (Figure 3b). The signal saturated after fifteen minutes, and the equilibrium signal change showed a linear concentration-dependence over a wide dynamic range (Figure 3c). Based on these findings, statistical analyses were performed to predict how the biosensor would work in clinical settings.

Inverse regression calculations demonstrated that the test’s limit of detection with 95% confidence was 20 ng of total protein from the cell lysate (Figure 3d). Influenza nucleoprotein represents only a small fraction of the total protein in the sample, so this value likely corresponds to picogram sensitivity. In addition to sensitivity, diagnostic tests must also be accurate in order to separate true positives from false positives. To predict test accuracy, a Receiver Operating Characteristic (ROC) curve was generated to describe the relationship between the number of true positives among diseased patients *vs.* the number of false positives among healthy subjects (Figure 3e). The estimation was based on a simulation of 2000 imaginary subjects, including 1,000 diseased patients and 1000 healthy subjects. By assuming that, on average, diseased patients have at least a five-times greater concentration of viral antigen than healthy subjects, the calculations yielded a predicted accuracy rate of 96%.

Beyond high sensitivity and accuracy, the ICS biosensor is unique within the immunodiagnostic field, because of its direct electronic readout and lack of processing steps. Indeed, all of the required components for detection and signal amplification are contained within the tethered bilayer, so addition of the target analyte is the only necessary step to yield a response. When compared to commonly used ELISA and immunochromatography tests in a prospective study by Oh *et al.*, the ICS biosensor demonstrated comparable levels of sensitivity and reproducibility for the analysis of clinical respiratory specimens from patients suspected to have influenza [43]. Notably, unlike the ICS biosensor, these standard methods require either lengthy pretreatment of sample or an internal control. In terms of detection sensitivity, the ELISA and immunochromatography tests did show higher endpoint sensitivities. This could be an issue for diagnoses at early stages of infection when viral loads are low, but there are also opportunities for improvement in the ICS biosensor. The antibody fragments presented on the tethered lipid bilayer are first generation versions, and the authors of the study reported that antibody maturation engineering is ongoing in order to increase affinity for viral antigen and, in turn, endpoint sensitivity. Beyond this example, protein engineering not only can enhance the capabilities of tethered bilayer platforms for specific applications, but also can create new sensor strategies not found in nature.

#### 3.1.2. Stochastic Sensor Arrays

Ion channels play an important role in communicating cell signals across membranes. Molecules that rapidly and reversibly bind to specific sites on ion channels are referred to as channel blockers and modulate ion channel conductance in order to transmit a signal. An excellent example is that of amiloride-sensitive sodium channels on the tongue’s surface, which mediate the taste of salt [44]. In addition to the biological implications of signaling pathways, the behavior of ion channels at the single molecule level can serve as the basis for stochastic sensing in engineered systems [45]. Through genetic manipulations, binding sites for a target analyte can be created on the ion channel, generally within the channel lumen. Under an applied potential, a measureable current flows through a single ion channel in the absence of analyte. When the target analyte reversibly binds to the ion channel, several changes in conductance occur, which, taken together, provide a wealth of information about the analyte. The frequency of conductance changes reveals analyte concentration, while the amplitude and mean duration of each binding event provides a characteristic signature for identification of the particular analyte species. 

**Figure 4:**
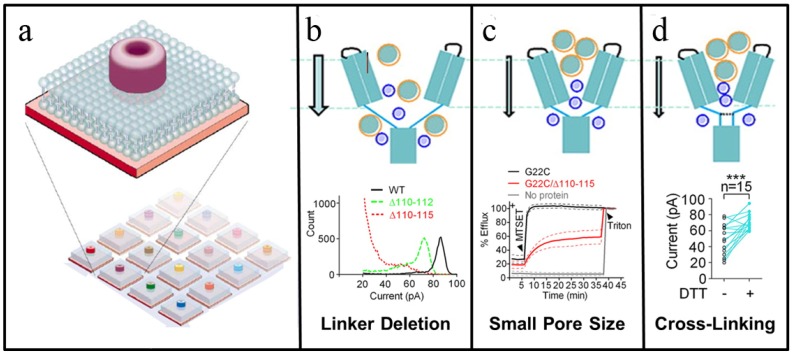
Stochastic sensing using engineered ion channels. (**a**) Conceptual design of an ion channel microarray for stochastic sensing. Each array spot contains a uniquely engineered ion channel pore that transmits a characteristic signal upon binding of the target analyte. (**b**) **Top:** Deletions in the TM2/CB linker region of MscL reduce amplitude of single channel conductance. **Bottom:** Current histogram of mutant MscL’s with TM2/CB linker deletions. Compared to the single channel current of wild type MscL, MscL Δ110-112 exhibits a clearly distinguished peak conductance. Amplitude of single channel current of MscL Δ110-115 is significantly smaller and has more heterogeneous characteristics. (**c**) **Top:** Rational design can decrease the pore size of MscL ion channel. **Bottom:** Calcein dye release as a function of time from lipid vesicles with different reconstituted MscL channels, as follows: G22C (black), G22C/Δ110-115 (red) or no MscL (gray). (**d**) **Top:** Introduction of three consecutive cysteines within MscL (A110-112C) induces cross-linking of channel subunits. **Bottom:** Recorded changes in the current of MscL A110-112C mutant before and after DTT treatment. Adapted with permission from [49]. Copyright 2012 American Chemical Society.

One of the most popular ion channels for stochastic sensing has been staphylococcal α-haemolysin (HL), in large part because it has a large single-channel conductance that is useful for biosensor applications. Moreover, X-ray crystallographic studies have revealed the channel’s structure at high resolution, and there are several opportunities within the channel for engineering analyte binding sites, including the channel pore and an extramembraneous domain that contains a large cavity. Through rational mutation and functionalization of these domains, α-HL has proven capable of sensitive detection of metal ions, organic molecules and macromolecules. Based on these early studies, there is interest in developing multiplexed stochastic sensor arrays that consist of families of related ion channels that mimic the mammalian olfactory system and its series of “crossreactive” receptors with varying affinities for analytes (Figure 4a). In order to realize such an array, tethered lipid bilayers are among the most promising platforms for stochastic sensor applications, because the solid support provides durability, as well as the capability, to micropattern bilayer patches comprised of different ion channels with individual electrode transducers [46].

Despite the promise of supported lipid bilayers for ion channel measurements, a long-standing challenge in the field has been to develop supported bilayers that have sufficient electrical sealing properties to record single-channel conductance. Andersson *et al.* reported the single-channel conductance measures of gramicidin pores using a thiolipid-based tethered bilayer platform [47]. Notably, the recorded conductance values were in good agreement with those obtained by tip-dip electrophysiological measurements. Interestingly, the tethered bilayer platform is also able to host the mechanosensitive (MscL) ion channel, which is gated by membrane tension [48]. While MscL channel conductance typically requires a combination of applied potential and pressure in patch-clamp experiments, only an applied potential was required for single-channel conductance recording in the tethered bilayer. For both types of measurements, computations showed that channel gating requires a membrane tension of 12 dyn/cm, and this finding supports that membrane stress rather than induced pressure is responsible for MscL channel gating.

More recently, Yang *et al.* reported the design of engineered MscL channels with tunable responses that may find application in stochastic sensors [49]. MscL is a homopentameric complex, and each subunit has two transmembrane α-helices (TM1 and TM2) and a single carboxyl terminal α-helix, the latter of which arranges to form a five-fold cytoplasmic bundle (CB). To decrease the pore size, amino acid deletions were made within the TM2/CB linker region. As shown in Figure 4b, wild-type MscL had a single channel current of 84.7 ± 1.7 pA compared to 73.1 ± 1.2 pA in truncated MscL Δ110–112. By contrast, the single channel current of another truncated form, MscL Δ110–115, was more heterogeneous and significantly smaller in magnitude, with most recordings below 30 pA. In addition, the linker deletion mutants were less sensitive to membrane tension, and this lower mechanosensitivity may prove advantageous, depending on the application. Since the MscL channel has been proposed to reset the gain in microchip devices that have reached saturation, this decreased sensitivity may serve to increase the time between resets by allowing greater build-up of ionic species in the reservoir between the solid support and lower leaflet of the bilayer before reaching the gate tension.

Another mutant of MscL that bears an amino acid substitution at G22C has been previously considered as a trigger for controlled release of liposomal payloads. Specifically, the positively charged, thiol-reactive compound MTSET can gate G22C MscL. When the G22C and Δ110–115 mutations were combined into a new MscL mutant, the channel exhibited slower translocation of larger compounds upon gating. To test the functional significance of this effect, different versions of MscL were reconstituted into liposomes loaded with calein dye. Upon the addition of MTSET, 100% calcein efflux occurred within a few minutes for liposomes containing G22C MscL, whereas calcein efflux reached less than 60% over 35 min for liposomes containing G22C/Δ110–115 MscL (Figure 4c). Thus, both mutants are sensitive to MTSET treatment, but have different release profiles. Similarly, the addition of three consecutive cysteines into the TM2/CB linker region of MscL A110–112C can cause cross-linking that reduces single channel conductance. When dithiothreitol (DTT) is added to cleave the disulfide bridges, the conductance of the MscL A110–112C channel increases (Figure 4d). Likewise, the introduction of histidine residues in the TM2/CB linker region can cause reversible changes in conductance based on zinc ion coordination. While mutant MscL channels have not yet been tested in tethered lipid bilayers, the recently demonstrated capability of tethered bilayers for stochastic sensing offers great promise for future studies based on these next-generation engineered pores. 

### 3.2. Air-Stable Bilayers

With the increasingly sophisticated sensing capabilities of tethered lipid bilayers, there has long been interest in developing rugged versions that support practical applications, such as clinical diagnostics and pharmaceutical drug screening, especially in high-throughput formats [50,51]. However, this goal has been difficult to attain, in large part because model membrane platforms are unstable when exposed to air [52]. While hybrid bilayers based on SAMs remain intact under dry conditions, they are not able to host transmembrane proteins. Alternative stabilization strategies based on polymerizable diacetylene lipids [53], surface coverage with proteins [54] or polymer brushes [55], and immersion in trehalose-containing solutions [56] have limited success for single rounds of drying, but can also interfere with biologically relevant parameters, such as membrane fluidity or cell surface receptor-ligand interactions. Recently, a new method for bilayer stabilization was reported by Deng *et al.* and allows the platform to be repeatedly dehydrated and rehydrated without detrimental effects on the platform’s ability to mimic natural properties of biological membranes [57].

**Figure 5:**
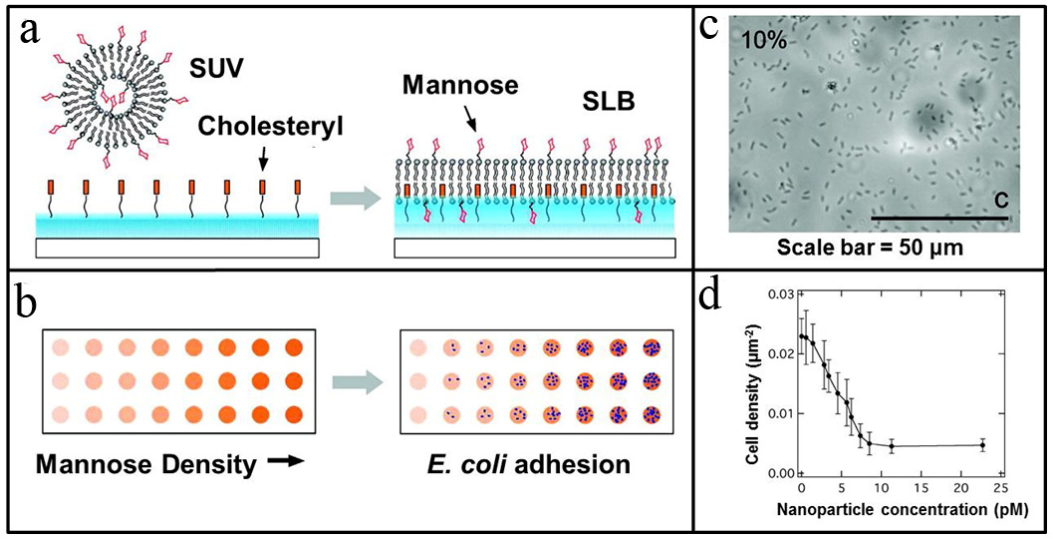
Air-stable tethered lipid bilayers for high-throughput biological measurement. (**a**) Self-assembly of a tethered lipid bilayer upon adsorption of unilamellar vesicles results in the presentation of mobile glycan receptors. (**b**) Engineering design strategies permit control of glycan density in order to optimize pathogen adhesion. (**c**) Optical microscopy image of *E. coli* ORN178 cells adsorbed on top of a tethered lipid bilayer containing 10 mol% mannose receptors. Adsorbed *E. coli* are depicted as elongated spots, as illustrated by blue arrows. Scale bars equals 50 μm. (**d**) The addition of mannose-presenting nanoparticles inhibits adsorption of ORN178 *E. coli* cells to the tethered lipid bilayer in a concentration-dependent manner. Adapted with permission from [61]. Copyright 2009 American Chemical Society.

The approach takes inspiration from cholesterol and its natural propensity to act as a stabilizing influence that modulates the physical properties of lipid membranes [58], including local conformational ordering and increased lipid packing density [59]. By covalently tethering a dispersed layer of cholesteryl molecules on a hydrophilic poly(ethyleneglycol) (PEG) polymer brush thin film, cholesteryl is evenly distributed and its surface density is controllable, depending on the reaction time of the immobilization step. After functionalizing the PEG brush with cholesteryl, the adsorption of lipid vesicles can promote the formation of a tethered bilayer that is uniformly supported by interactions with cholesteryl groups across the entire bottom leaflet (Figure 5a). To achieve a stabilizing effect, the cholesteryl surface density must be at least 0.3 mol·nm^−2^, and this value corresponds to a cholesteryl-to-lipid ratio of 1:6 in the bottom leaflet. Test experiments confirmed that the bilayer remained intact for at least ten cycles of dehydration and rehydration. Phospholipid lateral mobility was also maintained in the range of 1.3 ± 0.2 µm^2^·s^−1^ throughout the cycling. From a manufacturing perspective, the air-stability of the tethered bilayer is also significant, because it permitted robotic spotting of nanoliter volume suspensions of lipid vesicles. Typically, larger volumes are needed due to the risk of evaporation and possible exposure to air that would disrupt the bilayer. With this risk no longer an issue, lower volume requirements could potentially drive down material costs and increase the prospects for high-throughput array production. 

As a next step, the authors tested the functional significance of the platform for peripheral and transmembrane protein studies. A small fraction of the glycolipid, ganglioside GM1, was incorporated into the membrane to monitor binding of cholera toxin B (CTB). One CTB pentamer binds five individual GM1 surface receptors, so this model system requires membrane fluidity in order to achieve sufficiently strong multivalent interactions for specific binding. Fluorescent microscopy imaging of FITC-labeled CTB confirmed binding to the tethered bilayer, and an IC_50_ value of 6 nM was obtained that is in good agreement with literature results [60]. Phospholamban, a single domain transmembrane protein, was also successfully reconstituted in the tethered bilayer and recognized by fluorescently labeled antibodies. Beyond these proof-of-concept examples, recent work has focused on translating the capabilities of this platform into a high-throughput array for analyzing multivalent cell surface interactions involving glycan receptors.

Zhu *et al.* have reported a study on the adhesion of *E. coli* bacterial cells to mannose cell surface receptors presented in the tethered lipid bilayer format [61]. By varying the surface density of mannose receptors, the number density of adhered *E. coli* cells from the ORN178 strain was modulated based on high affinity binding of cell lectins to the mannose receptors (Figure 5b). As shown in Figure 5c, optical microscopy images reveal the oblong, rod shape of *E. coli* cells adhered upon a tethered lipid bilayer containing 10% mannose. To confirm that adhesion was dependent on specific multivalent binding to the mannose receptors, a negative control cell strain, *E. coli* ORN208, was also tested. Since there is a point mutation in the lectin binding domain of this strain, this strain has greatly reduced affinity to mannose receptors. As such, adhesion of the ORN208 strain saturated at 0.001 cells·µm^−2^ *vs.* 0.03 cells·µm^−2^ for the ORN178 strain. Based on these studies, two different types of adhesion were identified, monovalent and multivalent adhesion, leading the authors to propose that dynamic clustering of simple glycans may simulate the functions of complex oligo-glycans. This finding is significant in two contexts. Technologically, the tethered lipid bilayer demonstrates the capability of fluidic glycan array to control glycan density over several orders of magnitude. Biologically, quantitative profiling of protein-glycan interactions enables the discovery of novel mechanisms to explain cell surface processes, such as innate immune response and cell-cell communication.

Beyond fundamental science, the tethered lipid bilayer also shows promise as a novel platform technology for drug discovery and development. In addition to profiling the interactions between *E. coli* cells and mannose cell surface receptors, Zhu *et al.* should be cited in the Ref explored inhibition of these interactions using glyco-mimetics [61]. Nanoparticles coated with mannose receptors were pre-incubated together with *E. coli* ORN178 cells before cell adhesion to a tethered lipid bilayer containing 7.5% mannose was monitored. As the nanoparticle concentration increased, the number density of adhered cells decreased (Figure 5d). This correlation indicates that the nanoparticles act as competitive inhibitors, and the IC_50_ value was determined to be 4 pM. Importantly, the mechanism of inhibition depends on a multivalent interaction between the mannose-presenting nanoparticles and the *E. coli* cells. By contrast, free mannose at concentrations as high as 1 mM had no inhibitory effect. Taken together with its air-stability and functional capabilities, the tethered lipid bilayer has broad potential as a platform for analyzing interactions that occur at cell surfaces. Indeed, glycolipids are just one class out of many types of surface receptors for which this platform can analyze ligand-receptor interactions, as well as identify competitive inhibitors in a high-throughput format.

## 4. Perspectives

From its early motivation to improve the stability of planar lipid bilayers on solid supports, the tethered bilayer field has evolved over the years to focus on the molecular engineering of lipid membranes with precisely tuned functions. These efforts have required highly cross-disciplinary approaches that bring together researchers from engineering, science and medicine to tackle different design needs. For example, creative organic syntheses have led to the design of novel thiolipid tethers that combine biologically significant components, such as branched hydrocarbon chains, from archaebacteria together with oligoethyleneoxy spacers and functional groups that promote covalent immobilization on a range of substrates, such as gold, silicon oxide and aluminum oxide. No less important has been the introduction of protein engineering strategies, which enable the creation of sensory elements, such as antibody fragments for optimized molecular recognition or ion channels with characteristic signal traces for highly sensitive detection of target analytes. 

Along with these design advances, there has been simultaneous expansion in the range of surface-sensitive analytical techniques that can be applied to characterize tethered lipid bilayers. Optical, acoustic and spectroscopic methods are now available to complement electrochemical detection, depending on the characterization needs, and can provide detailed information about molecular binding, environment and orientation. The effects of these increasing capabilities have not only benefited fundamental science. In the mid-2000’s, a joint-venture company called Biosensor Enterprises was formed between Dow Corning and Genencor in order to commercialize the ICS biosensor for point-of-care analyte detection. A handheld biosensor with a disposable 16-well cartridge was produced, but the technology has not yet reached the point of mass manufacturing. Nevertheless, future commercial opportunities in the field are promising. Commercially available tethered lipid bilayer kits were recently introduced by two biotechnology companies, SDx Tethered Membranes and Microsurfaces, Inc.

Looking forward, there is also ample opportunity to expand the range of applications for tethered lipid bilayers. Several proof-of-concept examples have demonstrated the utility of these platforms for studying transmembrane proteins, and yet, to date, there has not been much translational research into drug discovery and development. Since over 60% of clinically approved drug targets are membrane-associated proteins, expanding work in the pharmaceutical sciences is an important goal for the field. Recent advances on multiplexed sensor arrays will support these efforts, because an important requirement for traditional drug discovery is high-throughput screening. From a design perspective, another future direction of the field will be quicker and more cost-effective methods to produce tethered lipid bilayers. As demonstrated by Deng *et al.*, the air-stability of cholesteryl-tethered bilayers is an excellent example of a design strategy that enables nanoliter sample volumes and robotic spotting [57]. Taken together, the design of tethered lipid bilayer has begun to resemble a molecular toolkit. From precise control over the chemical and physical properties of sensor elements. such as tethers, phospholipids and ion channels, to sensitive detection capabilities, we are beginning to develop an integrated framework to rationally design tethered lipid bilayers for an increasingly wide range of biotechnology applications. 
